# Inhaled nitric oxide preserves ventricular function during resuscitation using a percutaneous mechanical circulatory support device in a porcine cardiac arrest model: an echocardiographic myocardial work analysis

**DOI:** 10.1186/s12872-021-01992-w

**Published:** 2021-04-17

**Authors:** Christoph Nix, Rashad Zayat, Andreas Ebeling, Andreas Goetzenich, Uma Chandrasekaran, Rolf Rossaint, Nima Hatam, Matthias Derwall

**Affiliations:** 1 Department of Anesthesiology, Medical Faculty, RWTH University Hospital Aachen, RWTH Aachen University, 52074 Aachen, Germany; 2 Abiomed Europe GmbH, Aachen, Germany; 3 Department of Thoracic and Cardiovascular Surgery, Medical Faculty, RWTH University Hospital Aachen, RWTH Aachen University, 52074 Aachen, Germany; 4 Abiomed, Inc., Danvers, MA USA

**Keywords:** Left ventricular assist device, Percutaneous mechanical circulatory support, Cardiac arrest, Cardiopulmonary resuscitation, Left ventricular unloading, Nitric oxide, Impella

## Abstract

**Background:**

Resuscitation using a percutaneous mechanical circulatory support device (iCPR) improves survival after cardiac arrest (CA). We hypothesized that the addition of inhaled nitric oxide (iNO) during iCPR might prove synergistic, leading to improved myocardial performance due to lowering of right ventricular (RV) afterload, left ventricular (LV) preload, and myocardial energetics. This study aimed to characterize the changes in LV and RV function and global myocardial work indices (GWI) following iCPR, both with and without iNO, using 2-D transesophageal echocardiography (TEE) and GWI evaluation as a novel non-invasive measurement.

**Methods:**

In 10 pigs, iCPR was initiated following electrically-induced CA and 10 min of untreated ventricular fibrillation (VF). Pigs were randomized to either 20 ppm (*20 ppm*, n = 5) or 0 ppm (*0 ppm*, n = 5) of iNO in addition to therapeutic hypothermia for 5 h following ROSC. All animals received TEE at five pre-specified time-points and invasive hemodynamic monitoring.

**Results:**

LV end-diastolic volume (LVEDV) increased significantly in both groups following CA. iCPR alone led to significant LV unloading at 5 h post-ROSC with LVEDV values reaching baseline values in both groups (*20 ppm:* 68.2 ± 2.7 vs. 70.8 ± 6.1 mL, *p* = 0.486; *0 ppm*: 70.8 ± 1.3 vs. 72.3 ± 4.2 mL, *p* = 0.813, respectively). LV global longitudinal strain (GLS) increased in both groups following CA. LV-GLS recovered significantly better in the 20 ppm group at 5 h post-ROSC (*20 ppm*: − 18 ± 3% vs. *0 ppm*: − 13 ± 2%, *p* = 0.025). LV-GWI decreased in both groups after CA with no difference between the groups. Within *0 ppm* group, LV-GWI decreased significantly at 5 h post-ROSC compared to baseline (1,125 ± 214 vs. 1,835 ± 305 mmHg%, *p* = 0.011). RV-GWI was higher in the *20 ppm* group at 3 h and 5 h post-ROSC (*20 ppm*: 189 ± 43 vs. *0 ppm*: 108 ± 22 mmHg%, *p* = 0.049 and *20 ppm*: 261 ± 54 vs. *0 ppm*: 152 ± 42 mmHg%, *p* = 0.041). The blood flow calculated by the Impella controller following iCPR initiation correlated well with the pulsed-wave Doppler (PWD) derived pulmonary flow (PWD vs. controller: 1.8 ± 0.2 vs. 1.9 ± 0.2L/min, *r* = 0.85, *p* = 0.012).

**Conclusions:**

iCPR after CA provided sufficient unloading and preservation of the LV systolic function by improving LV-GWI recovery. The addition of iNO to iCPR enabled better preservation of the RV-function as determined by better RV-GWI. Additionally, Impella-derived flow provided an accurate measure of total flow during iCPR.

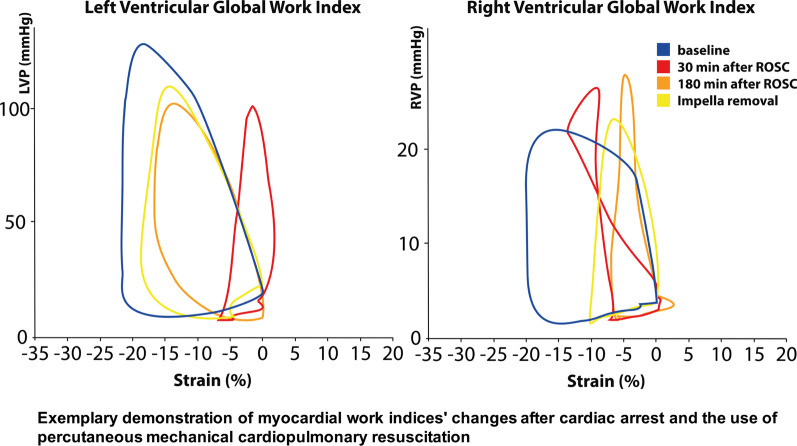

## Background

Cardiac arrest (CA) remains associated with high mortality and poor functional outcome. Despite the growing clinical use of mechanical circulatory support (MCS) for cardio-pulmonary resuscitation (CPR) after CA, the contribution towards a higher survival rate and the better functional outcome is yet to be demonstrated. An animal study showed a beneficial effect of the early use of a percutaneous mechanical circulatory support device, termed intravascular CPR (iCPR), on post-CPR survival [1]. An additional inhaled nitric oxide (iNO) application in an animal model resulted in improved trans-pulmonary blood flow and was associated with improved neurological outcomes [2]. We hypothesized that the synergistic effects of iNO during iCPR were a result of improved myocardial performance due to lowering of right ventricular (RV) afterload, left ventricular (LV) preload, and myocardial energetics. Previous studies have demonstrated a cardio-protective effect of ventricular unloading by percutaneous MCS through reduction in myocardial wall stress and myocardial work (MW) [3]. Recently, echocardiographic MW assessment has emerged as a non-invasive method to evaluate myocardial performance and gain more insights into myocardial energetics and mechanics, given the strong correlation with invasive pressure–volume (PV) measurements [4].

Thus, in the present study, we aimed to better understand the effect of iNO during iCPR on the myocardial performance and mechanics in a well-established large animal model of CA [1, 2]. The left (LV) and right ventricular (RV) function before, during, and after iCPR were analyzed using 2-D transesophageal echocardiography (TEE), deformation imaging, tissue Doppler (TDI) and non-invasive myocardial work measurement. In addition, trans-pulmonary flow (TPF) inferred by pulsed-wave Doppler (PWD) during iCPR was compared to the calculated pump flow derived from the Impella controller.

## Methods

The experimental protocol was approved by the appropriate governmental institution (Landesamt für Natur, Umwelt und Verbraucherschutz NRW (LANUV), Recklinghausen, Germany) and has been previously described [1, 2]. All animals received adequate care according to the precepts of the Helsinki declaration. Ten healthy female swine (Deutsche Landrasse, Sus scrofa domesticus), approximately four months of age, and weighing 44 to 57 kg were used in this study. Animals were sourced from the Institute for Laboratory Animal Research, University Hospital RWTH Aachen, Aachen, Germany. All pigs had a clinical examination on arrival at the facility. Animals were housed in pens with a 12 h-day-night cycle and access to drinking water ad libitum. Twelve hours before the experiment, the pigs were set on nil per os except for drinking water access. The experimental procedures were carried out in accordance with the ARRIVE guidelines 2.0 (Animal Research: Reporting of In Vivo Experiments) [5].

### Animal instrumentation

**Fig. 1:**
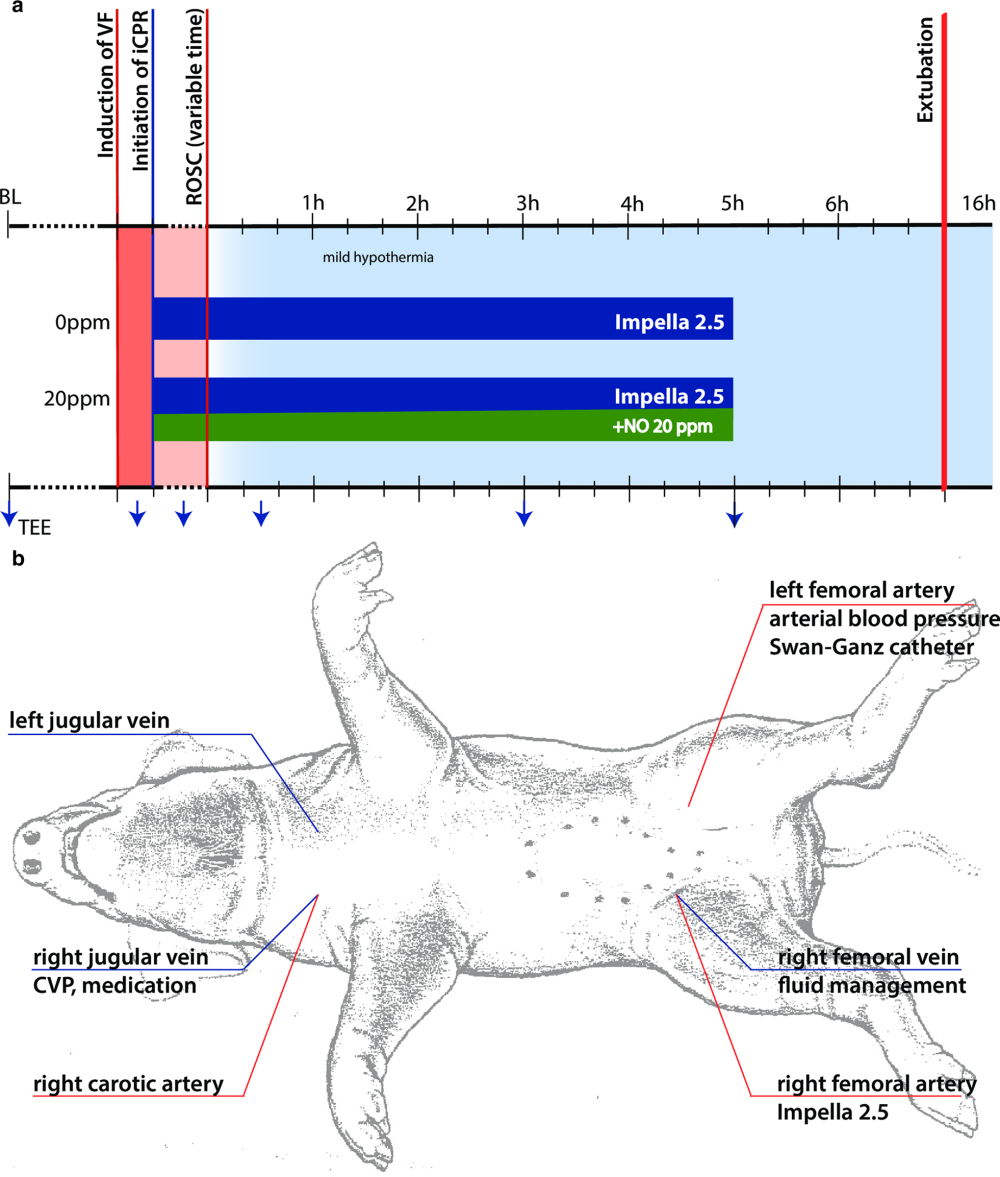
Experimental model (**a**) and instrumentation (**b**). CVP: central venous pressure; iCPR: Percutaneous mechanical cardiopulmonary resuscitation; VF: ventricular fibrillation; ROSC: Return of spontaneous circulation

Figure 1 provides and overview of animal instrumentation and the experiment model. The pigs were weighed before the start of the experiment. General anesthesia was induced by intramuscular injection of 4 mg/kg azaperone (Stresnil, Janssen-Cilag GmbH, Neuss, Germany), followed by intravenous injection of 15 mg/kg sodium pentobarbital (Narcoren, Boehringer Ingelheim Vetmedica GmbH, Ingelheim am Rhein, Germany). Anesthesia was maintained by continuous intravenous sodium pentobarbital infusion at a rate of 4 mg/kg/h. The pigs were placed in a supine position, intubated orotracheally, and the legs were fixed in an extended position. Animals were ventilated with an inspired oxygen fraction of 0.3 (Servo Ventilator 300A; Siemens AG, Munich, Germany). Tidal volume was set to 10 ml/kg, and respiratory rate was adjusted to keep the end-tidal carbon dioxide partial pressure within a physiologic range (35 ± 4 mmHg). A continuous five lead electrocardiogram (ECG) and pulse oximetry were performed. Convective air heating was used to maintain body temperature at 38 ± 0.5 °C during preparation (Warm Touch 5200; Tyco Healthcare, Pleasanton, CA, USA).

### Hemodynamic monitoring

Arterial blood pressure was obtained using a fluid-filled catheter (Vygon, Ecquen, France) placed into the left femoral artery. For pulmonary artery pressure and cardiac output measurements, a Swan-Ganz catheter (744HF75; Edwards Lifesciences, Irvine, CA, USA) was flow-directed into the pulmonary artery and connected to a cardiac output monitor (Vigilance; Edward Lifesciences). Impella flow was taken from the motor current based flow calculation on the AIC (Automated Impella Controller) as described in detail earlier [6].

### Cardiac arrest model

As described previously [1, 2], cardiac arrest was induced using an alternating current of 1 to 2 mA delivered to the endocardium of the right ventricle, resulting in VF. Simultaneously, mechanical ventilation was discontinued.

A 13-French sheath introducer (Impella 2.5 introducer kit 13 F, 13 cm; Abiomed Europe GmbH, Aachen, Germany) was placed in the right femoral artery. A modified Impella 2.5 left ventricular assist device (Abiomed) equipped with a shortened angled cannula to meet the anatomical constraints of the animal was introduced using the vascular access into the left ventricle with the help of fluoroscopy guidance, pigtail catheter (Cordis 6 F PIG 145° 110 cm Super Torque Plus; Cordis, Miami Lakes, FL, USA) and a guidewire (Platinum Plus 0.018 in × 260 cm; Boston Scientific, Natick, MA, USA) (see Fig. 1 for details). Impella 2.5 support was activated at the maximum achievable flow ten minutes following the onset of VF. The pig was then randomized (closed envelope method) to a FiO_2_ of 1.0 without any added iNO (group *0 ppm*; n = 5) versus a FiO_2_ of 1.0 blended with 20 ppm of iNO (group *20 ppm*; n = 5). Therapeutic mild hypothermia was induced using 0.5L of a 6 °C cold Ringer’s solution and topical application of ice bags to the groins in all animals. The blood temperature was maintained at 33 ± 1 °C. A continuous infusion of Ringer’s solution was administered at 4 ml/kg/h to ensure adequate hydration.

### Post cardiac arrest care

As previously described by our group [1, 2], after ROSC and weaning from the respirator, animals were brought to their cages and monitored permanently by research staff. Within 48 hr. after weaning from respirator, animals were euthanized by intravenous injection of a lethal dose of pentobarbital.

### Transesophageal Echocardiography

All TEE studies were performed by the same board-certified physician at baseline (after all catheters were placed), during VF and iCPR, and at 30mins, 3 h, 5 h post-ROSC. We used a commercially available ultrasound machine (Vivid E9, GE Vingmed Ultrasound AS, Horten, Norway) equipped with a 6VT-D [3.0–8.0 MHz] TEE probe. Our detailed TEE protocol in swine has been described previously [7]. In brief, at least three consecutive cardiac cycles were stored and averaged for all measurements. The analysis was carried out offline using the device´s software package (EchoPAC Version 202; GE Vingmed Ultrasound AS, Horten, Norway) according to the recommendation of the European Association of Cardiovascular imaging [8]. Images were acquired from mid-esophageal, trans-gastric, and upper esophageal views. Valvular pathologies were excluded by color-Doppler. LV end-diastolic (LVEDV) and end-systolic (LVESV) volumes were measured in the mid-esophageal four-chamber (4-CH) views and deep esophageal two-chamber (2-CH) views. The modified biplane Simpson’s method was used for Ejection fraction (EF) measurements [9]. RV fractional area change (RV-FAC) was determined according to the following formula:

$${\text{RV-FAC }} = \frac{{{\text{End-diastolic}}\;{\text{area}} - {\text{end-systolic}}\;{\text{area}}}}{{{\text{End-diastolic}}\;{\text{area}}}} \times {\text{100}}\%$$

TDI was used to measure RV peak systolic tricuspid annulus velocity (TASV) from a 4-CH view as an indicator of RV longitudinal function [10]. The PWD sample was placed in RV- (RVOT) and LV-outflow tracts (LVOT) to obtain velocity time integrals (VTI). RV Cardiac output (CO) was calculated using the formula: CO = VTI_RVOT_ x RVOT cross-sectional area x heart rate and for the LV CO = VTI_LVOT_ x LVOT cross-sectional area x heart rate.

### Speckle tracking and non-invasive myocardial work measurements:

2-D speckle-tracking (STE) was used to define LV global longitudinal strain (LV-GLS) and RV-GLS. LV GLS was measured using the three apical chamber views (4-CH, 2-CH and apical long axis (LAX)). For STE analysis of the RV the 4CH view for the RV anterior wall was used. The RV free wall was captured from a modified upper long axis view (LAX) of the RV in- and outflow tract and part of the posterior RVOT was obtained from a mid-esophageal LAX RV view (Please refer to supplementary Fig. 1 for a representative view). Aortic and mitral valve opening and closure times were determined by PWD. The systolic arterial and pulmonary blood pressures were entered into the EchoPAC software (GE Vingmed Ultrasound AS, Horten, Norway). After completing the STE analysis of LV and RV, EchoPAC calculated pressure-strain loops (PSL). Adjusted LV pressure curves corresponding to the length of isovolumic and ejection phases were generated by EchoPAC software, as described by Russel et al. [11, 12]. The global myocardial work index (GWI) was calculated from the area within the PSL and the following parameters were then calculated from the PSL: 1) GWI: The total myocardial work from mitral valve closure to mitral valve opening is represented by the total area of PSL; 2) global constructive myocardial work (GCW): the ventricular work which contributes to the ejection during systole: GCW = positive work during systole + negative work during isovolumic relaxation; 3) global wasted myocardial work (GWW): the ventricular work that does not contribute to the ejection of blood. GWW = negative work during systole + positive work during isovolumic relaxation; 4) global myocardial work efficiency (GWE): the fraction of constructive myocardial work to total work [11–13]. LV pressure was estimated by adjusting a reference pressure curve with measured blood pressure and with echocardiography derived valvular event timing [11–13]. We used the blood pressure taken from the femoral artery for that purpose. RV's pressure was measured using the Swan-Ganz catheter. The myocardial work was expressed in mmHg%. EchoPAC provided a validated assessment of LV myocardial work indices. To our knowledge, measurements for RV myocardial work indices with EchoPAC were not validated by GE healthcare or by any other research group yet.

### Statistical analysis

Statistical analysis was performed with STATA IC16 (StataCorp., TX, USA) and Jamovi project (Version 1.6.1, https://www.jamovi.org). Figures were created using Graph Pad Prism version 7.0a for MAC OS X (Graph Pad Software, La Jolla, California USA) and prepared for submission using Adobe® Illustrator® CS6 (Adobe Inc., San Jose, California, USA). The normal distribution of continuous variables was assessed using the Shapiro–Wilk-Test, and was expressed as mean ± standard deviation. A mixed-effects model was used for between-group comparisons at different time-points and Sidak’s test was used to account for multiple comparisons. The non-parametric Friedman-test and Dunn’s correction for multiple comparisons was used for intergroup comparisons at different time points. Pearson’s correlation test was performed to assess the correlation between the invasive thermodilution measured CO (CO_TD_) and the PWD derived CO (CO_PWD_). Adjusted p-values for multiple comparisons are presented and *p* < 0.05 was considered statistically significant.

## Results

### Impella controller derived flow correlates well with pulsed-wave Doppler derived flow

All 10 pigs survived 15 h post-ROSC and were successfully extubated. No complications due to TEE probe insertion or during TEE image acquisition were observed. Body surface area was similar between groups (*0 ppm*: 1.2 ± 0.1 vs. *20 ppm*: 1.3 ± 0.1 m^2^, *p* = 0.278). At least two different clear views of 4-CH, 2-CH, and LAX at all given time points were obtained in all animals.

**Fig. 2:**
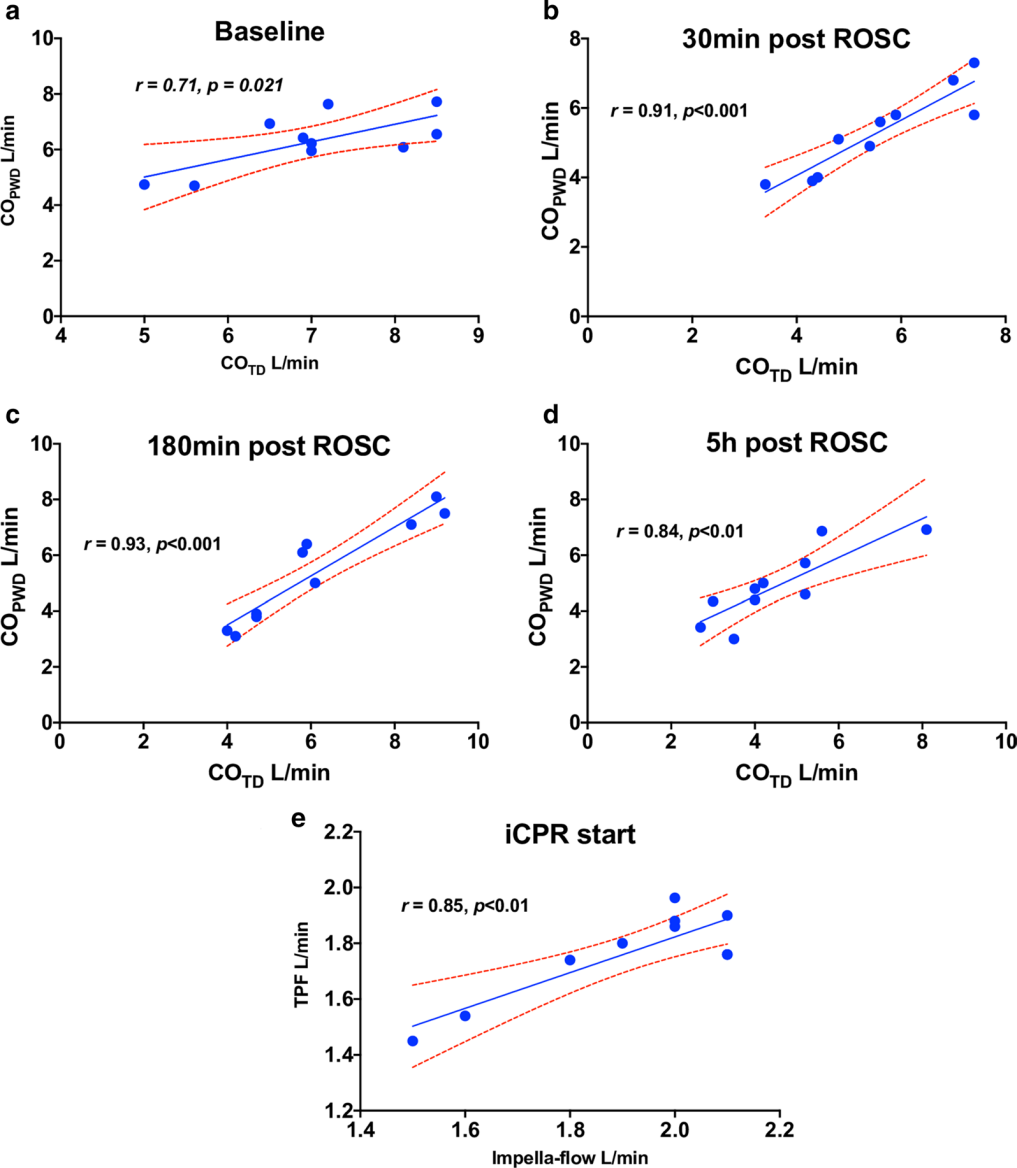
Cardiac output correlation between different methods of measurement. Comparison between cardiac output derived from pulsed-wave Doppler vs. thermodilution at different time-points (**a**-**d**) or vs. Impella flow as shown on the automated Impella Controller during iCPR (**e**) in all animals (not separated by treatment group). Shown are the individual comparisons, linear correlation within a 95% confidence interval (red dotted line)

The mean heart rate at baseline was 92 ± 14 min^−1^. The systemic and pulmonary artery pressure at baseline did not differ between groups and are presented in Table 1. CO_PWD_ correlated well with the invasively measured CO_TD_ at baseline, 30 min, 3 h and 5 h (*r* = 0.71, *p* = 0.021; *r* = 0.91, *p* < 0.001; *r* = 0.93, *p* < 0.001; *r* = 0.84, *p* < 0.01, respectively) (Fig. 2). The calculated pump flow displayed by the automated Impella controller correlated well with the PWD derived pulmonary flow following iCPR initiation (1.8 ± 0.2 vs. 1.9 ± 0.2 L/min, *r* = 0.85, *p* = 0.012).

**Table 1:** Hemodynamic parameters before, during and after the resuscitation using a percutaneous mechanical circulatory support device

	0 ppm	20 ppm	Total	p
BSA m^2^	1.2 ± 0.1	1.3 ± 0.1	1.3 ± 0.1	0.271
**Baseline**				
HR min^−1^	85.6 ± 13.9	98.4 ± 11.6	92.0 ± 13.8	0.152
SBP mmHg	129.2 ± 16.5	126.4 ± 20.3	127.8 ± 17.5	0.817
DBP mmHg	68.6 ± 8.6	67.4 ± 5.9	68.0 ± 7.0	0.805
SPAP mmHg	26.6 ± 2.8	24.8 ± 3.6	25.7 ± 3.2	0.400
DPAP mmHg	14.2 ± 2.7	12.4 ± 2.9	13.3 ± 2.8	0.337
CO_PWD_ L/min	6.9 ± 1.7	7.0 ± 0.8	7.0 ± 1.3	0.878
CO_TD_ L/min	6.7 ± 1.5	7.3 ± 0.7	7.0 ± 1.2	0.459
**Cardiac arrest**				
CO_PWD_ L/min	1.7 ± 0.2	1.8 ± 0.2	1.8 ± 0.2	0.341
Impella flow L/min	1.9 ± 0.3	1.9 ± 0.2	1.9 ± 0.2	0.892
HR_pp-VF_ min^−1^	20.8 ± 1.1	21.4 ± 0.8	21.1 ± 0.9	0.491
**30 min post-ROSC**				
HR min^−1^	115 ± 24.3	130 ± 30	128 ± 36	0.055
SBP mmHg	95.2 ± 12.9	95.6 ± 25.1	95.4 ± 18.8	0.976
DBP mmHg	64.8 ± 10.6	64.4 ± 10.9	64.6 ± 10.1	0.955
SPAP mmHg	26.2 ± 3.8	25.2 ± 6.2	25.7 ± 4.9	0.766
DPAP mmHg	14.4 ± 3.5	11.8 ± 5.3	13.1 ± 4.5	0.388
CO_TD_ L/min	5.5 ± 1.7	5.6 ± 1.2	5.6 ± 1.4	0.900
CO_PWD_ L/min	5.1 ± 1.1	5.7 ± 1.2	5.4 ± 1.1	0.433
**3 h post-ROSC**				
HR min^−1^	102.8 ± 13.9	111.6 ± 21.6	107.2 ± 17.7	0.466
SBP mmHg	99.6 ± 8.6	114.2 ± 28.0	106.9 ± 21.0	0.298
DBP mmHg	68.0 ± 7.3	65.0 ± 11.7	66.5 ± 9.3	0.639
SPAP mmHg	28.0 ± 5.5	28.4 ± 4.6	28.2 ± 4.8	0.903
DPAP mmHg	15.0 ± 3.2	14.4 ± 3.6	14.7 ± 3.3	0.790
CO_PWD_ L/min_	4.6 ± 1.0	6.6 ± 1.1	5.7 ± 1.5	0.016
CO_TD_ L/min	5.1 ± 0.8	6.8 ± 2.3	5.9 ± 1.8	0.172
**5 h post-ROSC**				
HR min^−1^	75.0 ± 11.0	104.8 ± 19.6	89.9 ± 21.7	0.018
SBP mmHg	110.2 ± 13.8	113.0 ± 18.0	111.6 ± 15.2	0.790
DBP mmHg	68.0 ± 9.8	66.2 ± 9.0	67.1 ± 8.9	0.771
SPAP mmHg	32.0 ± 4.4	23.0 ± 1.0	27.5 ± 5.6	0.008
DPAP mmHg	16.2 ± 3.7	11.8 ± 1.3	14.0 ± 3.5	0.037
CO_PWD_ L/min_	4.0 ± 0.6	5.8 ± 1.0	4.9 ± 1.2	0.036
CO_TD_ L/min	3.5 ± 0.6	5.6 ± 1.5	4.5 ± 1.6	0.021

CO_*PWD*_: Pulsed- wave Doppler derived cardiac output; CO_*TD*_: Cardiac output measured with thermodilution method using wan-Ganz catheter; DBP: Diastolic blood pressure; DPAP: Diastolic pulmonary artery pressure; HR: Heart rate; HR _*pp-VF*_: Peak to peak ventilation frequency as the Heart rate during cardiac arrest; SBP: Systolic blood pressure; SPAP: Systolic pulmonary artery pressure

### Arterial blood gas analysis

**Fig. 3:**
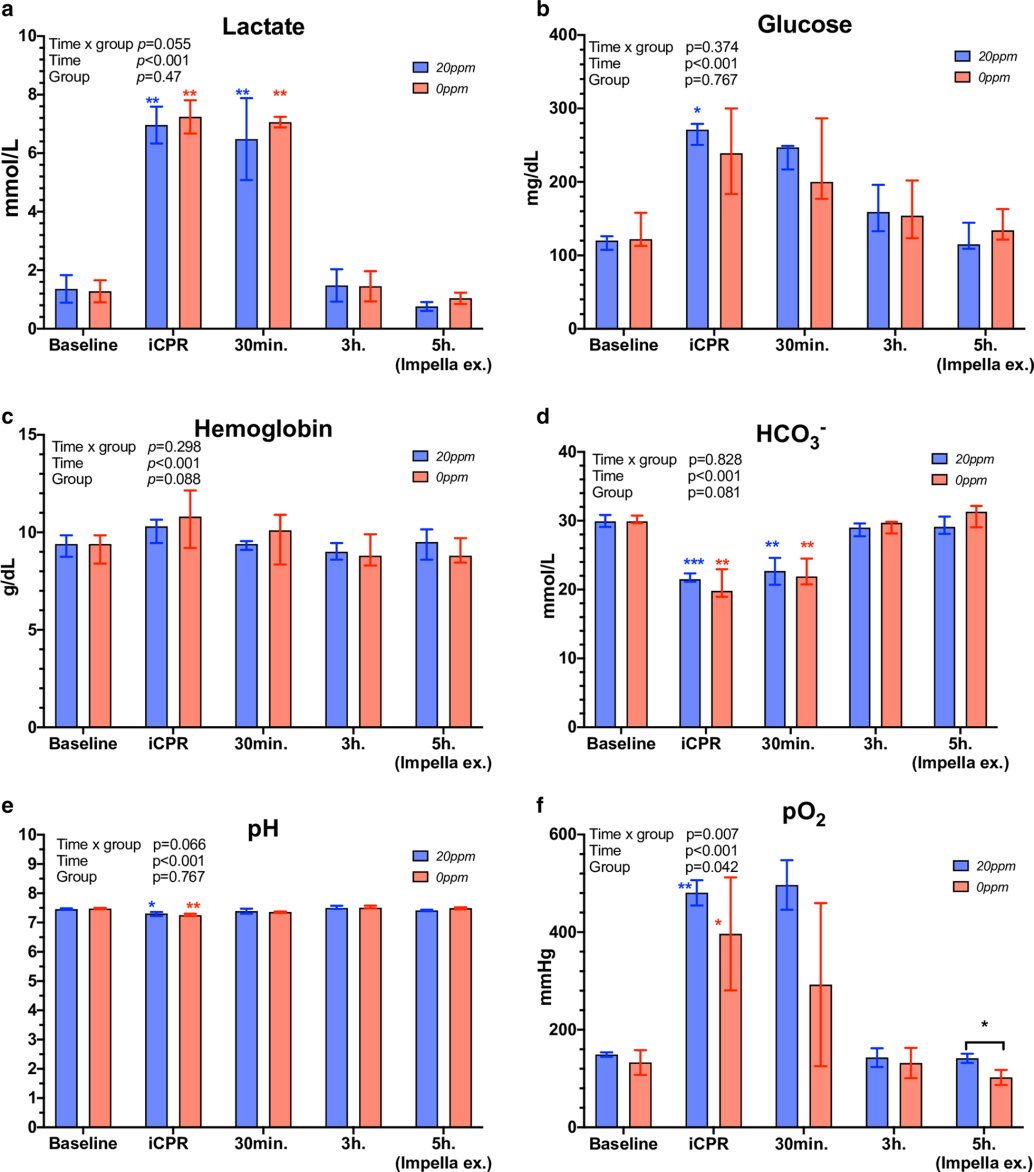
Time-course of arterial blood gas analysis parameters. **a** Lactate mmol/L, **b** Glucose mg/dL; **c** hemoglobin g/dL; **d** hydrogen carbonate (HCO_3_^−^) mmol/L; **e** power of hydrogen (PH); **f** arterial partial pressure of oxygen (pO_2_). *p < 0.05; **p < 0.01; ***p < 0.001. Red asterisk: *0 ppm* group compared to baseline; blue asterisk: *20 ppm* group compared to baseline; black asterisk: between groups at specific time-point

An overview of arterial blood gas analysis (BGA) throughout the experiment is provided in Fig. 3 and Table 2. At baseline, all BGA parameters did not differ between the two groups. Lactate increased significantly in both groups during iCPR and 30 min following ROSC (20* ppm*: baseline vs. iCPR: 1.4 ± 0.5 vs. 7.0 ± 0.6 mmol/L, *p* = 0.005; 0* ppm:* Baseline vs iCPR*:* 1.3 ± 0.4 vs. 7.2 ± 0.6 mmol/L, *p* < 0.001) and returned to baseline value 3 h post-ROSC. During and after iCPR, all BGA parameters, with exception of pO_2_ and pCO_2_, did not differ between the two groups at all measured time-points. At 5 h post-ROSC, the pO_2_ was significantly higher in the 20* ppm* group than in the 0* ppm* group (141.4 ± 9.4 vs. 102.4 ± 15.4 mmHg, *p* = 0.011), and the pCO_2_ was significantly lower in the 20* ppm* compared to the 0* ppm* group (46.1 ± 1.9 vs. 38.7 ± 2.7 mmHg, *p* = 0.007).

**Table 2:** Arterial blood gas analysis before, during and after resuscitation

	0 ppm	20 ppm	total	p
**Baseline**				
Lactate mmol/L	1.3 ± 0.4	1.4 ± 0.5	1.3 ± 0.4	1.000
Hemoglobin g/dL	9.2 ± 0.8	9.3 ± 0.6	9.2 ± 0.7	0.834
Glucose mg/dL	132.8 ± 24.6	117.4 ± 9.7	125.1 ± 19.4	0.465
pH	7.48 ± 0.02	7.46 ± 0.01	7.47 ± 0.02	0.251
HCO_3_ mmoL	30.1 ± 0.6	30.0 ± 1.1	30.1 ± 0.8	0.751
BE mmoL	6.1 ± 0.8	6.1 ± 1.1	6.1 ± 0.9	0.600
pO_2_ mmHg	132.8 ± 25.4	149.2 ± 4.4	141.0 ± 19.2	0.251
pCO_2_ mmHg	43.3 ± 2.4	39.8 ± 2.4	41.5 ± 2.9	0.057
**iCPR**				
Lactate mmol/L	7.2 ± 0.6	7.0 ± 0.6	7.1 ± 0.6	0.402
Hemoglobin g/dL	10.7 ± 1.5	10.1 ± 0.7	10.4 ± 1.1	0.465
Glucose mg/dL	241.2 ± 63.2	266.0 ± 17.6	253.6 ± 45.6	0.754
pH	7.26 ± 0.03	7.29 ± 0.07	7.27 ± 0.05	0.347
HCO_3_ mmoL	20.7 ± 2.1	21.7 ± 0.6	21.2 ± 1.5	0.602
BE mmoL	− 4.1 ± 2.4	− 3.6 ± 1.6	− 3.8 ± 1.9	0.917
pO_2_ mmHg	396.4 ± 115.7	480.6 ± 25.9	438.5 ± 90.7	0.117
pCO_2_ mmHg	47.3 ± 8.3	50.4 ± 4.0	48.8 ± 6.3	0.465
**30 min post ROSC**				
Lactate mmol/L	7.1 ± 0.2	6.5 ± 1.4	6.8 ± 1.0	0.116
Hemoglobin g/dL	9.7 ± 1.3	9.3 ± 0.3	9.5 ± 0.9	0.600
Glucose mg/dL	225.4 ± 57.3	235.8 ± 20.2	230.6 ± 40.9	0.602
pH	7.39 ± 0.07	7.35 ± 0.1	7.37 ± 0.07	0.142
HCO_3_ mmoL	22.5 ± 1.9	22.7 ± 2.3	22.6 ± 2.0	0.754
BE mmoL	− 2.5 ± 1.7	− 1.6 ± 2.7	− 2.1 ± 2.2	0.463
pO_2_ mmHg	292.4 ± 167.1	496.6 ± 50.7	394.5 ± 158.5	**0.028**
pCO_2_ mmHg	39.4 ± 9.3	41.8 ± 2.5	40.6 ± 6.5	0.602
**3 h post ROSC**				
Lactate mmol/L	1.4 ± 0.5	1.5 ± 0.6	1.5 ± 0.5	0.530
Hemoglobin g/dL	9.0 ± 0.8	9.0 ± 0.4	9.0 ± 0.6	0.917
Glucose mg/dL	161.0 ± 51.1	163.4 ± 37.4	162.2 ± 42.3	0.602
pH	7.53 ± 0.05	7.51 ± 0.05	7.52 ± 0.05	0.465
HCO_3_ mmoL	29.1 ± 1.0	28.7 ± 1.2	28.9 ± 1.1	0.402
BE mmoL	4.9 ± 1.2	4.8 ± 1.4	4.8 ± 1.2	1.000
pO_2_ mmHg	131.7 ± 31.1	143.0 ± 19.2	137.4 ± 25.1	0.402
pCO_2_ mmHg	34.4 ± 5.6	33.3 ± 5.6	33.9 ± 5.3	0.917
**5 h post ROSC**				
Lactate mmol/L	1.0 ± 0.2	0.8 ± 0.2	0.9 ± 0.2	0.139
Hemoglobin g/dL	9.0 ± 0.8	9.4 ± 0.9	9.2 ± 0.8	0.401
Glucose mg/dL	140.6 ± 22.1	124.4 ± 24.4	132.5 ± 23.6	0.249
pH	7.49 ± 0.02	7.42 ± 0.03	7.45 ± 0.04	0.050
HCO_3_ mmoL	30.7 ± 1.7	29.3 ± 1.3	30.0 ± 1.6	0.175
BE mmoL	7.2 ± 1.7	6.0 ± 1.6	6.6 ± 1.7	0.251
pO_2_ mmHg	102.4 ± 15.4	141.4 ± 9.4	121.9 ± 23.9	**0.010**
pCO_2_ mmHg	46.1 ± 1.9	38.7 ± 2.7	42.4 ± 4.4	**0.007**

BE: Base excess mmol/L; HCO3^−^: Hydrogen carbonate mmol/L; pH: Power of Hydrogen; pO_2_: arterial partial pressure of oxygen mm Hg; pCO_2_: arterial partial pressure of carbon dioxide mmHg

### Application of iNO significantly increases cardiac output and improves left ventricular systolic function recovery

**Fig. 4:**
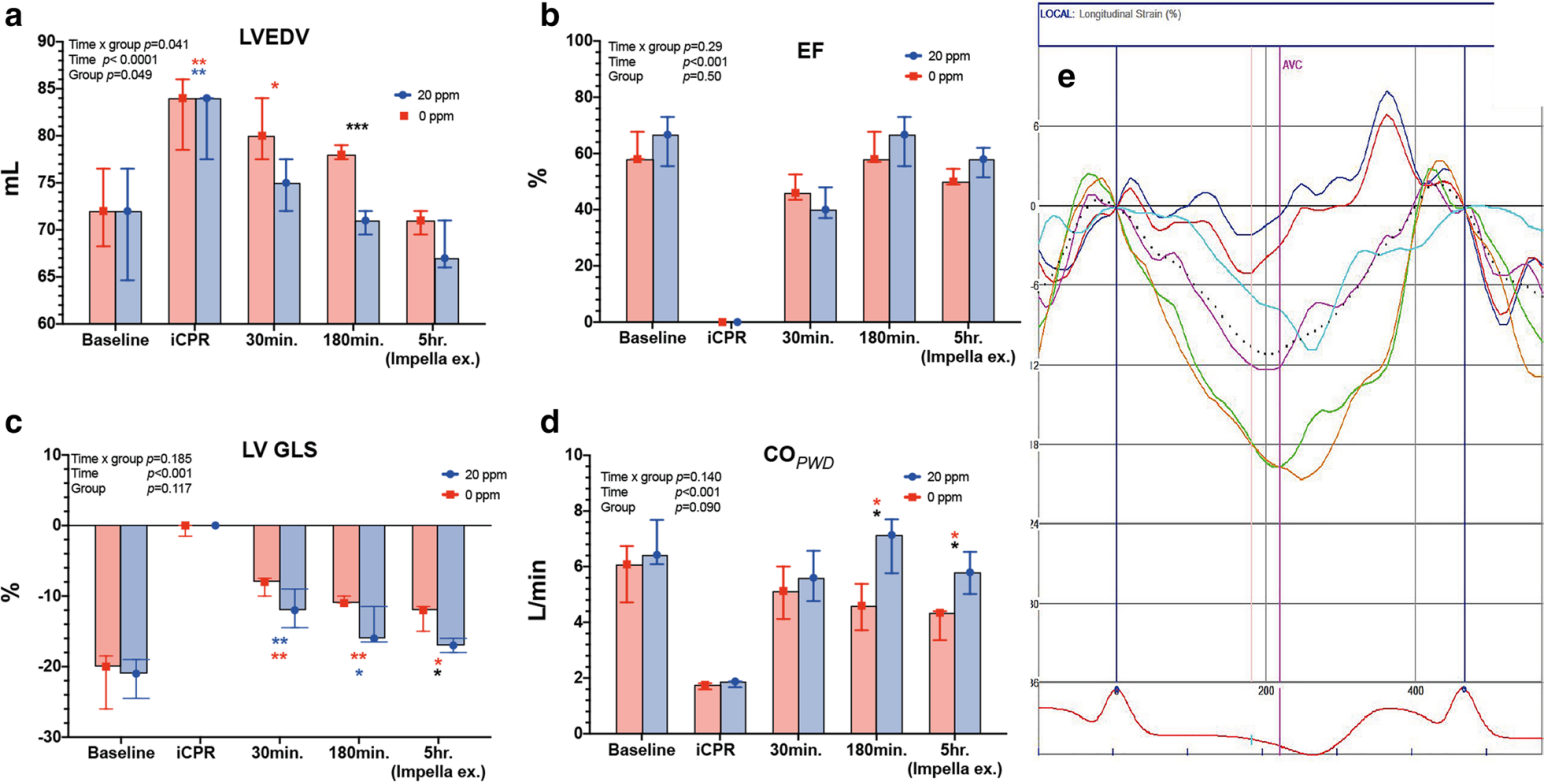
Time-course of left ventricular 2-dimensional echocardiographic parameters. **a** Left ventricular end-diastolic volume (LVEDV); **b** left ventricular ejection fraction (EF); **c** left ventricular global longitudinal strain (LV GLS); **d** cardiac output as measured by pulsed-wave Doppler (CO_*pwd*_); **e **Example of regional longitudinal strain analysis of the left ventricle. *p < 0.05; **p < 0.01; ***p < 0.001. Red asterisk: *0 ppm* group compared to baseline; blue asterisk: *20 ppm* group compared to baseline; Black asterisk: between groups at specific time-point

At baseline, LVEDV, LV-EF and LV-GLS did not differ between groups. After induction of VF, LVEDV increased significantly in both groups compared to baseline (Fig. 4a). Over the course of iCPR for 5 h, LVEDV decreased and returned closer to baseline values in both groups (Fig. 4a and Table 3). However, LVEDV in the 20 ppm group was higher at all time points and was statistically significant at 3 h post-ROSC (*0 ppm*: 78.2 ± 1.1 vs. *20 ppm*: 70.8 ± 1.3 mL, *p* < 0.001). LV-EF did not differ significantly between groups at all time points or within each group compared to baseline (Fig. 4b). The LV-GLS improved throughout the duration of iCPR in both groups from baseline (Fig. 4c). The LV-GLS values were significantly lower at 3 h and 5 h post-ROSC in the *20 ppm* group compared to the *0 ppm* group (5 h: *0 ppm*: − 13.1 ± 2.4 vs. *20 ppm*: − 18.2 ± 3.4, *p* = 0.025) (Fig. 4c). The CO decreased in both groups at iCPR initiation and improved over the course of iCPR. The CO_PWD_ increased significantly in the *20 ppm* group at 3 h and 5 h post-ROSC compared to the *0 ppm* group (3 h: *0 ppm*: 4.6 ± 0.9 L/min vs. *20 ppm*: 6.6 ± 1.1, *p* = 0.016; 5 h: *0 ppm*: 4.0 ± 0.6 L/min vs. *20 ppm*: 5.8 ± 1.0, *p* = 0.036) (Fig. 4d).

**Table 3:** Comparison of echocardiographic parameters between the groups with and without inhaled nitric oxide

	0 ppm	20 ppm	Total	p
**Baseline**				
RVD1 cm	3.1 ± 0.4	3.4 ± 0.4	3.3 ± 0.4	0.332
RVD2 cm	2.6 ± 0.2	2.7 ± 0.4	2.6 ± 0.3	0.417
RVD3 cm	4.0 ± 0.2	3.8 ± 0.4	3.9 ± 0.3	0.424
RV-FAC %	44.1 ± 6.0	42.9 ± 6.6	43.5 ± 6.0	0.772
LVEDV mL	70.5 ± 3.5	70.9 ± 6.1	70.7 ± 4.7	0.911
LVESV mL	31.9 ± 4.6	33.4 ± 3.6	32.6 ± 4.0	0.583
LV-EF %	61.5 ± 5.8	64.7 ± 9.2	63.1 ± 7.5	0.525
LV-GLS %	− 21.0 ± 5.1	− 21.6 ± 3.4	− 21.3 ± 4.1	0.833
LV-GWI mmHg%	1835.3 ± 306.2	1751 ± 270.2	3444.3 ± 5242.8	0.336
LV-GCW mmHg%	2095.8 ± 527.4	2103.2 ± 405.8	2099.5 ± 443.6	0.981
LV-GWW mmHg%	368.0 ± 208.8	352.4 ± 115.9	360.2 ± 159.4	0.887
LV-GWE %	85.2 ± 4.7	80.0 ± 1.4	82.6 ± 4.2	0.054
TASV cm/sec	13.6 ± 1.1	13.8 ± 1.5	13.7 ± 1.3	0.817
RV-GLS %	− 16.4 ± 3.2	− 14.4 ± 1.8	− 15.4 ± 2.7	0.267
RV-GWI mmHg%	338.2 ± 45.3	286.8 ± 57.5	312.5 ± 55.8	0.155
RV-GCW mmHg%	376.4 ± 92.6	266.6 ± 41.1	321.5 ± 89.0	0.082
RV-GWW mmHg%	61.6 ± 13.8	55.4 ± 10.6	58.5 ± 12.1	0.949
RV-GWE %	79.8 ± 10.2	80.8 ± 6.1	80.3 ± 7.9	0.855
**Cardiac arrest**				
RVD1 cm	4.0 ± 0.3	4.2 ± 0.3	4.1 ± 0.3	0.397
RVD2 cm	3.3 ± 0.6	3.5 ± 0.4	3.4 ± 0.5	0.581
RVD3 cm	4.5 ± 0.2	4.6 ± 0.5	4.6 ± 0.4	0.749
RVEDA cm^2^	13.9 ± 1.6	15.2 ± 1.1	14.6 ± 1.4	0.183
LVEDV mL	81.0 ± 4.9	76.6 ± 7.5	78.8 ± 6.4	0.306
**30 min. post-ROSC**				
RVD1 cm	3.7 ± 0.3	3.6 ± 0.4	3.7 ± 0.3	0.582
RVD2 cm	3.0 ± 0.4	2.9 ± 0.4	2.9 ± 0.4	0.716
RVD3 cm	4.5 ± 0.2	4.6 ± 0.1	4.5 ± 0.2	0.776
RV-FAC %	27.4 ± 2.8	32.0 ± 9.3	30.5 ± 6.5	0.494
LVEDV mL	77.0 ± 6.5	73.8 ± 9.7	75.4 ± 8.0	0.558
LVESV mL	35.8 ± 5.1	35.4 ± 3.7	35.6 ± 4.2	0.897
LVEF %	47.6 ± 5.0	42.0 ± 8.1	44.8 ± 7.0	0.225
LV-GLS %	− 11.6 ± 3.3	− 13.2 ± 4.7	− 12.4 ± 3.9	0.548
LV-GWI mmHg%	891.2 ± 412.7	1201.8 ± 400.5	1046.5 ± 416.8	0.262
LV-GCW mmHg%	719.0 ± 497.8	919.6 ± 638.2	819.3 ± 549.8	0.595
LV-GWW mmHg%	360.4 ± 271.1	240.2 ± 148.1	300.3 ± 215.4	0.416
LV-GWE %	63.4 ± 7.7	75.0 ± 8.7	69.2 ± 9.9	0.256
TASV cm/sec	10.4 ± 4.9	9.8 ± 1.8	10.1 ± 3.5	0.803
RV-GLS %	− 6.6 ± 3.3	− 9.4 ± 2.9	− 8.0 ± 3.3	0.193
RV-GWI mmHg%	101.6 ± 34.5	92.0 ± 33.3	96.8 ± 32.4	0.666
RV-GCW mmHg%	152.2 ± 15.5	159.6 ± 20.2	164.1 ± 40.0	0.395
RV-GWW mmHg%	59.4 ± 11.9	49.4 ± 9.1	52.3 ± 13.2	0.843
RV-GWE %	70.6 ± 12.0	74.0 ± 8.6	72.3 ± 10.0	0.621
**3 h. post-ROSC**				
RVD1 cm	3.3 ± 0.3	3.4 ± 0.3	3.4 ± 0.3	0.782
RVD2 cm	2.7 ± 0.3	2.8 ± 0.1	2.8 ± 0.2	0.347
RVD3 cm	4.4 ± 0.1	4.3 ± 0.3	4.3 ± 0.2	0.718
RV-FAC %	32.5 ± 5.0	43.3 ± 7.0	43.2 ± 6.8	0.945
LVEDV mL	78.2 ± 1.1	70.8 ± 1.3	74.5 ± 4.1	< 0.001
LVESV mL	38.0 ± 4.9	35.2 ± 3.5	36.6 ± 4.3	0.342
LVEF %	61.5 ± 5.8	64.7 ± 9.2	63.1 ± 7.5	0.525
LV-GLS %	− 12.2 ± 2.8	− 16.0 ± 2.3	− 14.1 ± 3.1	0.058
LV-GWI mmHg%	896.2 ± 129.3	1399.2 ± 347.3	1147.7 ± 362.4	0.116
LV-GCW mmHg%	719.0 ± 497.8	1598.8 ± 849.2	1158.9 ± 803.5	0.081
LV-GWW mmHg%	264.6 ± 78.9	293.4 ± 212.5	279.0 ± 151.9	0.784
LV-GWE %	67.2 ± 3.3	81.4 ± 5.6	74.3 ± 8.7	0.010
TASV cm/sec	7.8 ± 1.6	11.4 ± 1.1	11.0 ± 1.5	0.024
RV-GLS %	− 10.0 ± 1.0	− 12.0 ± 2.7	− 11.0 ± 2.2	0.164
RV-GWI mmHg%	108.4 ± 22.6	189.6 ± 43.6	149.0 ± 53.9	0.049
RV-GCW mmHg%	203.4 ± 39.6	221.2 ± 85.6	212.3 ± 63.6	0.684
RV-GWW mmHg%	69.6 ± 11.9	47.4 ± 6.7	47.8 ± 13.3	0.049
RV-GWE %	72.2 ± 14.0	75.0 ± 11.1	73.6 ± 12.0	0.734
**5 h post-ROSC**				
RVD1 cm	3.5 ± 0.5	3.9 ± 0.7	3.7 ± 0.6	0.308
RVD2 cm	2.8 ± 0.4	3.2 ± 0.7	3.0 ± 0.6	0.287
RVD3 cm	4.4 ± 0.2	4.6 ± 0.1	4.5 ± 0.2	0.024
RV-FAC %	33.2 ± 4.3	41.1 ± 6.5	37.1 ± 6.7	0.053
LVEDV mL	70.8 ± 1.3	68.2 ± 2.7	69.5 ± 2.4	0.087
LVESV mL	38.0 ± 6.9	35.6 ± 4.5	36.8 ± 5.6	0.525
LVEF %	51.4 ± 3.0	57.0 ± 6.2	54.2 ± 5.5	0.108
LV-GLS %	− 13.0 ± 2.4	− 18.2 ± 3.4	− 15.6 ± 3.9	0.036
LV-GWI mmHg%	1125 ± 214	1529.2 ± 274.5	1251.3 ± 348.4	0.052
LV-GCW mmHg%	1203.8 ± 253.0	1728.4 ± 466.7	1466.1 ± 449.1	0.058
LV-GWW mmHg%	395.4 ± 159.4	367.0 ± 87.2	381.2 ± 122.1	0.736
LV-GWE %	75.8 ± 2.3	82.6 ± 4.0	79.2 ± 4.7	0.071
TASV cm/s	9.4 ± 1.3	12.8 ± 1.1	9.9 ± 2.2	0.012
RV-GLS %	− 9.0 ± 1.4	− 13.2 ± 2.2	− 11.1 ± 2.8	0.040
RV-GWI mmHg%	152.6 ± 42.4	261.6 ± 54.2	207.1 ± 73.5	0.041
RV-GCW mmHg%	224.4 ± 158.7	212.6 ± 53.8	218.5 ± 111.9	0.879
RV-GWW mmHg%	77.8 ± 14.8	50.2 ± 8.2	61.4 ± 22.4	0.046
RV-GWE %	68 ± 5.7	80 ± 5.2	76 ± 5.4	0.042

GCW: Global constructed myocardial work; GLS: Global longitudinal strain; GWE: Global myocardial work efficiency; GWI: Global myocardial work index; GWW: Global wasted work; LV: Left ventricle; LVEDV: Left ventricle end-diastolic volume; LVEF: Left ventricular ejection fraction; LVESV: Left ventricular end-systolic volume; RVEDA: right ventricle end-diastolic area; RVD1: right ventricle basal diameter from apical 4 chamber view; RVD2: right ventricular mid diameter at midlevel from apical 4 chamber view; RVD3: right ventricle longitudinal diameter; RV-FAC: right ventricular fractional area change; TASV: TASV: Tricuspid annular systolic velocity

### Application of iNO enhances right ventricular systolic function recovery

**Fig. 5:**
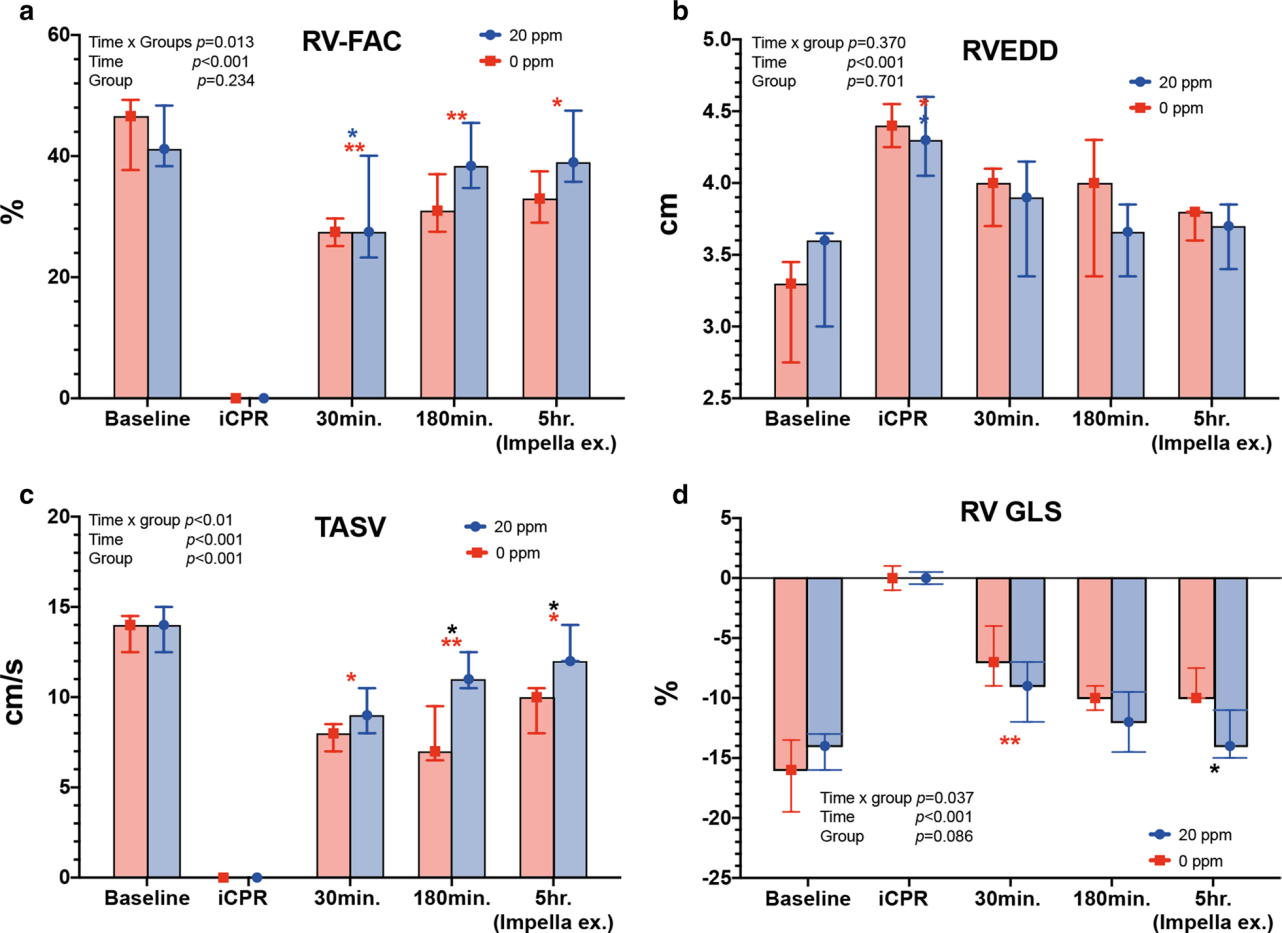
Time-course of right ventricular 2-dimensional echocardiographic parameters. **a** Right ventricular fractional area change (RV-FAC); **b** Right ventricular end-diastolic diameter (RVEDD); **c** Tricuspid annular systolic velocity (TASV); **d** right ventricular global longitudinal strain (RV GLS). *p < 0.05; **p < 0.01. Red asterisk: *0 ppm* group compared to baseline; blue asterisk: *20 ppm* group compared to baseline; black asterisk: between groups at specific time-point

RV echocardiographic measurements, RV basal diameters at end-diastole (RVEDD), RV-FAC, TASV and RV-GLS, did not differ between the two groups at baseline (Fig. 5a–d and Table 3). However, RV-FAC decreased significantly during CA and 30 min post-ROSC. In the *0 ppm* group, RV-FAC was lower at all time-points compared to baseline (0 ppm baseline vs. 30 min, vs. 3 h vs. 5 h post-ROSC: 44.1 ± 6.1% vs. 27.4 ± 2.8%, *p* = 0.004; vs. 32 ± 5%, *p* = 0.009; vs. 33.2 ± 4.3%, *p* = 0.001; see also Table 3). In the *20 ppm* group, RV-FAC improved at 3 h and recovered to baseline values at 5 h post-ROSC (baseline: 42.9 ± 6.6% vs. 5 h: 41.1 ± 6.4; *p* = 0.442) (Fig. 5a). RVEDD increased significantly in both groups at iCPR initiation, and slowly recovered over time but did not differ between groups (Fig. 5b and Table 3). Within the *0 ppm* group, TASV decreased significantly after CA and remained lower during the course of iCPR (baseline vs. 3 h: 13.6 ± 1.1 vs. 7.8 ± 1.6 cm/s, *p* = 0.024; baseline vs. 5 h: 13.6 ± 1.1 vs. 9.4 ± 1.3 cm/s, *p* = 0.009, respectively) (Fig. 5a and Table 3). The TASV values in the *20 ppm* group were higher at 3 h and 5 h post-ROSC compared to the *0 ppm* group (*20 ppm* vs. *0 ppm* at 3 h: 11.4 ± 1.1 vs. 7.8 ± 1.6 cm/s, *p* = 0.024; at 5 h: 12.8 ± 1.1 vs. 9.4 ± 1.3 cm/s, *p* = 0.012). The RV-GLS values were similar between the two groups at baseline and post-ROSC. However, RV-GLS at 5 h post-ROSC was significantly lower in the *20 ppm* group than 0 ppm (*0 ppm* vs. *20 ppm* at 5 h: − 9 ± 1.4% vs. − 13 ± 2.2%; *p* = 0.007; Fig. 5d).

### LV myocardial work indices indicate recovery during and after ventricular unloading

**Fig. 6:**
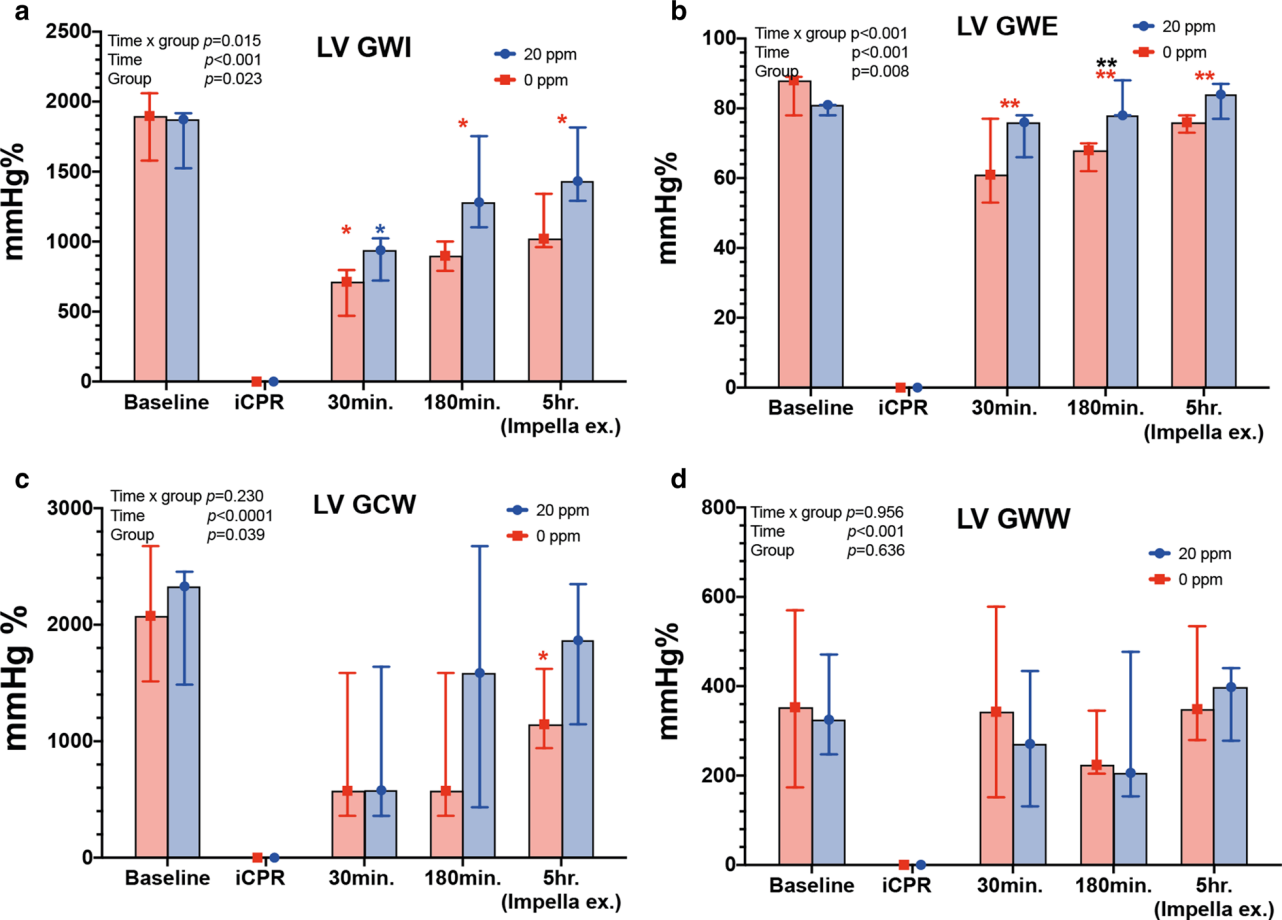
Changes of left ventricular function and myocardial work. **a** Left ventricular global work index (LV GWI); **b** Left ventricular global work efficiency (LV GWE); **c** Left ventricular global constructed work (LV GCW); **d** Left ventricular global wasted work (LV GWW). *p < 0.05; **p < 0.01. Red asterisk: *0 ppm* group compared to baseline; blue asterisk: *20 ppm* group compared to baseline; black asterisk: between groups at specific time-point

At baseline, myocardial work indices (GWI, GWE, GCW, GWW) did not differ between the two groups (Fig. 6a-d and Table 3). The LV-GWI did not differ between the two groups at any time. However, LV-GWI in the *20 ppm* group recovered after an initial decrease and began to recover at 30 min post-ROSC to reach almost baseline values at 5 h post-ROSC (baseline: 1,751 ± 270 mmHg% vs. 5 h: 1,529 ± 274 mmHg%, *p* = 0.401) (Fig. 6a and Table 3), while in the *0 ppm* group, LV-GWI was significantly lower compared to baseline at all time-points (baseline: 1,835 ± 305 mmHg%; 30 min: 891.2 ± 412 mmHg% (*p* = 0.032), 3 h: 896 ± 129 mmHg% (*p* = 0.011), and 5 h: 1,125 ± 214 mmHg% (*p* = 0.011); Fig. 6a). LV-GWE and LV-GCW had a similar time-course to LV-GWI and did not recover in the *0 ppm* group (Fig. 6b). The LV-GWW did not differ between groups and did not increase significantly in both groups at all time-points compared to baseline (Fig. 6c).

### RV myocardial indices indicate a more complex pattern and slower recovery

**Fig. 7:**
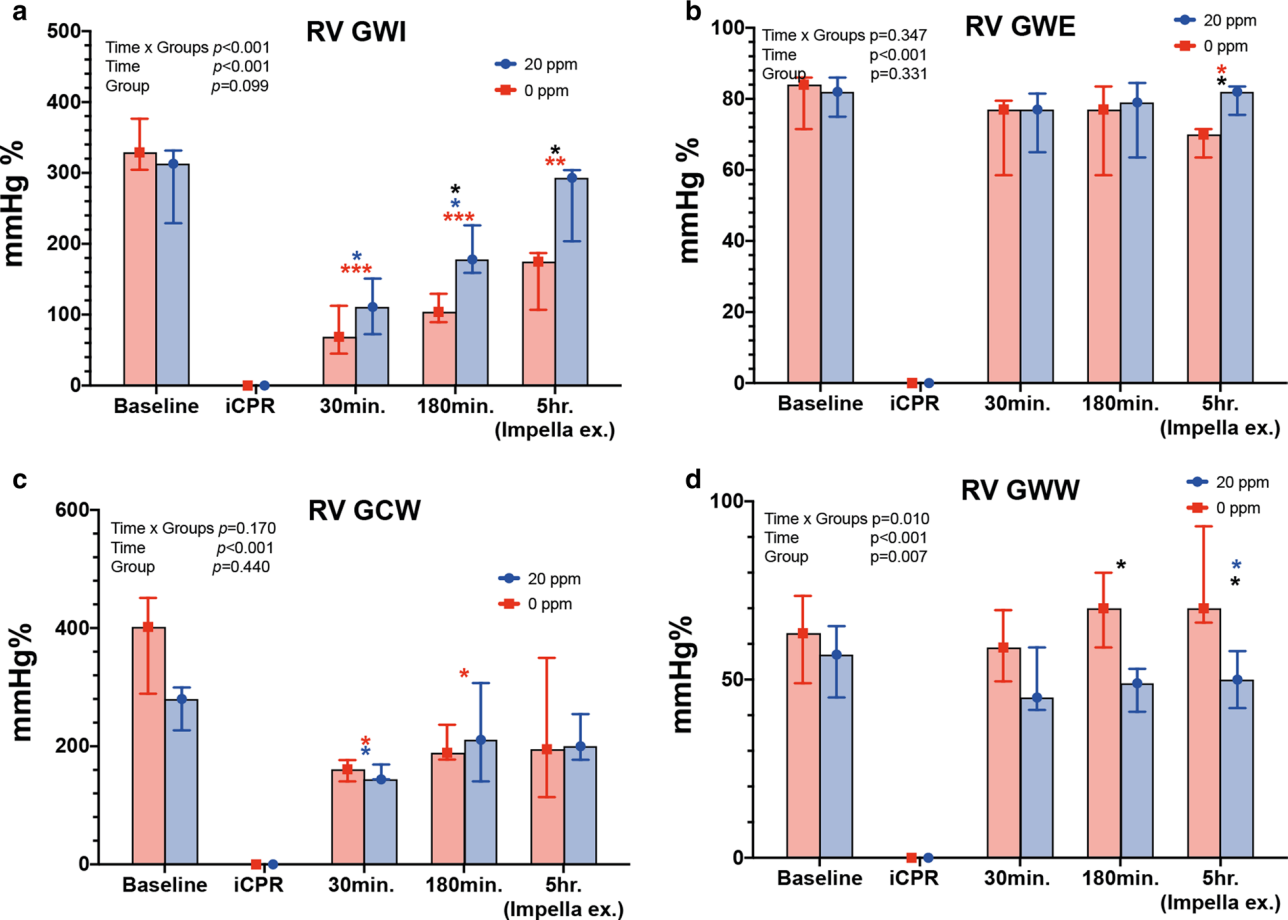
Changes of right ventricular function and myocardial work. **a** Right ventricular global work index (RV GWI); **b** Right ventricular global work efficiency (RV GWE); **c** Right ventricular global constructed work (RV GCW); **d** Right ventricular global wasted work (RV GWW). *p < 0.05; **p < 0.01; ***p < 0.001. red asterisk: *0 ppm* group compared to baseline; blue asterisk: *20 ppm* group compared to baseline; black asterisk: between groups at specific time-point

At baseline, the RV myocardial work indices did not differ between groups (*0 ppm* vs. *20 ppm*, Fig. 7a–c and Table 3). At 3 h and 5 h post-ROSC, RV-GWI was significantly lower in the *0 ppm* group compared to the *20 ppm* group (*0 ppm*: 108.4 ± 22.6 vs. *20 ppm*: 189.6 ± 43.6 mmHg%, *p* = 0.049 and *0 ppm*: 152.6 ± 42.4 vs. 261.6 ± 54.2 mmHg%, *p* = 0.041, respectively). Within the *0 ppm* group, the RV-GWI did not recover and remained significantly decreased at 30 min, 3 h and 5 h post-ROSC compared to baseline (baseline: 338.2 ± 45.3 mmHg% vs. 30 min: 101.6 ± 34.5 mmHg%, *p* < 0.001; vs. 3 h: 108.4 ± 22.6 mmHg%, *p* < 0.001; vs. 5 h: 152.6 ± 42.4 mmHg%, *p* = 0.012, respectively; Fig. 7b). In both groups, the RV-GCW decreased significantly after CA, but a slow recovery over time was noted in both groups with no difference between groups (Fig. 7b and Table 3). The RV-GWW increased significantly in the *0 ppm* group compared to the *20 ppm* group at 3 h and 5 h post-ROSC: 69.6 ± 11.9 vs. 47.4 ± 6.7, *p* = 0.049 and 77.8 ± 14.8 vs. 50.2 ± 8.2, *p* = 0.046, respectively (Fig. 7c). The RV-GWE increased significantly in the *20 ppm* group at 5 h post-ROSC compared to *the 0 ppm* group (80 ± 5.2 vs. 73 ± 5.7, *p* = 0.042, respectively).

### Diagnostic utilization of pressure-strain loops

**Fig. 8:**
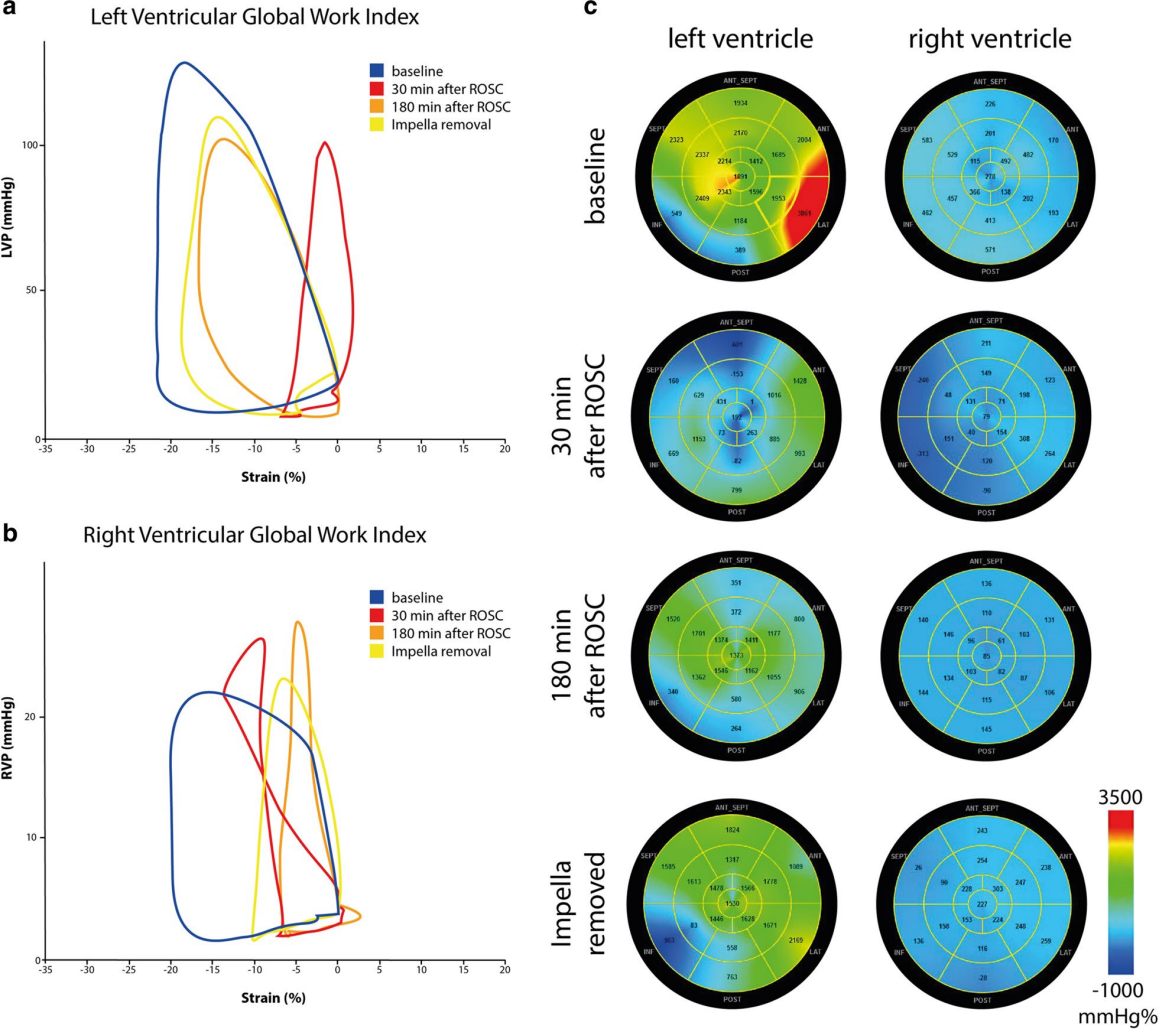
Exemplary demonstration of global myocardial work index changes during the experiment. **a**, **b** relation of left/right ventricular pressure to global left/right ventricular strain over time. Comparable to a PV-loop, this allows to gain insights on myocardial workload and function. **c** example of spatial distribution of right and left ventricular myocardial work index. Myocardial work indices are color coded from red to blue, where red color indicate the maximum value and blue the lowest myocardial work index value

Figure 8a is a representative overlay of the PS-loops of the LV. An acute injury following iCPR initiation is demonstrated by narrowing of the PS-loop (a sign of loss in maximally generated strain), thus explaining the ventricle´s inability to generate sufficient pressure [14–18].

The partially positive strain indicates a passive distention of the myocardium due to volume loading [16–18]. Unloading leads to slow recovery and progressive normalization of the PS-loop, which remains below (i.e., less negative) normal values but shows a curve pattern comparable to normal conditions [18, 19].

Figure 8b is a representative overlay of the PS-loops of the right ventricle. A pumping failure leads to increased RVP, and a loss in strain translates to a more complex strain-pressure relation [20, 21]. The RV strain reduction remains pronounced even after a longer duration of left ventricular support [21, 22].

Figure 8c demonstrates the spatial distribution of strain at different time-points. This allows differentiation of regional wall motion deterioration e.g., in acute myocardial infarction from global failure, thus providing a detailed perspective on myocardial recovery over time.

## Discussion

Resuscitation following cardiac arrest remains associated with significant mortality and morbidity [23]. Although treatment guidelines recommend inotropes and vasopressors, many studies have demonstrated their detrimental side effects [23]. In the early phase of resuscitation, treatments aim to augment or stabilize cardiac output, allowing minimal end-organ perfusion, and reducing or preventing hypoxic/ischemic injury [12–14]. However, these treatments cause increased myocardial stress and oxygen demand when oxygen supply is low [24, 25].

Trans-valvular mechanical support devices like the Impella device family uncouple the myocardial workload from the systemic cardiac output (which is then primarily provided by the pump), leading to adequate end-organ perfusion while resting the heart [3, 26]. Previous studies [27–29] have demonstrated the efficacy of this intravascular resuscitation, and the present study in an animal model confirms the results and provides additional insights on the temporal evolution of the effect.

### Global myocardial work and the principle of unloading

The pressure-strain (PS) loop generated by tracking the global myocardial strain vs. the LVEDP/RVEDP over the cardiac cycle provides a momentary impression of the myocardial work and is comparable to the pressure–volume (PV) loops derived from conductance catheters [4, 11, 12]. Russel et al. [11, 12] demonstrated a strong correlation between the non-invasive LV PS-loop area and the invasive PV-loop area. The LV PV-loop analysis considers the LV load and has been shown in experimental studies to be a reliable and robust method for quantifying LV performance. Moreover, the PV-loop area provides valuable information on the myocardial O_2_-consumption [30, 31]. Despite being a valuable and reliable method, the PV-loop method is not used in clinical practice due to its invasiveness [11, 12, 32, 33].

Evaluation of ventricular function is an essential component of all echocardiographic examinations. Despite the many LVEF measurement limitations, LVEF remains the most widely used parameter for systolic function evaluation [34]. More recently, the peak longitudinal strain from 2D-speckle tracking analysis has gained acceptance in the clinical routine. Compared to EF, GLS can detect subclinical myocardial abnormalities. However, GLS analysis suffers from load dependency and has limitations [35].

The PS-loop analysis incorporates both the deformation changes and afterload, thus overcoming the limitations of load dependency [4]. Therefore, GWI provides incremental information to EF and GLS analysis. While the PV-loop area reflects myocardial metabolic demand and oxygen consumption, the non-invasive myocardial work method provides myocardial energetics [11, 12, 33]. For several decades, stroke work evaluation played a key role in heart failure characterization and decision-making regarding optimal treatment. Traditional stroke work measurements are calculated using the LV PV-loop area derived from invasive catheterization [30, 31]. The PS-loop method has a robust correlation with invasive PV-loops and enables evaluating myocardial wasted work and work efficiency [12].

The value and clinical implications of non-invasively estimated myocardial work indices have been tested in several clinical entities. Recently, Galli et al. [36] demonstrated the value of GWW and GWI in providing details on dyssynchronous contraction and segmental work. The authors [36] identified, that responders to cardiac resynchronization therapy have a higher GCW than non-responders. Using GWI and GWW enabled the early identification of patients with acute coronary occlusion with non-ST-elevation [4]. Ischemia induces changes in myocardial contraction, and it has been shown that patients with subclinical ischemia have a dyssynchronous contraction and increased GWW [15].

Secondary right ventricular failure can aggravate cardiogenic shock [37, 38]. Thus, prevention of right ventricular distension might play a crucial role in limiting progression to right ventricular failure, even during intravascular resuscitation [38]. Notably, the right ventricular preload is increased during left ventricular mechanical support [39]. Transvalvular LV pumps depend on left ventricular preload to prevent suction and allow for optimal flows [39, 40]. Reduction in right ventricular afterload by pulmonary vasodilatation, leading to a decrease in pulmonary vascular resistance, should facilitate the reduction of left ventricular filling and right ventricular myocardial workload [41, 42]. The results of this study confirmed that iNO successfully decreases right ventricular afterload, normalizes RV filling pressure over time, and shifts the RV strain towards a normal (i.e., baseline) configuration.

### Further application perspectives of the GWI

The PS-loop method may enable an accurate ventricular-arterial coupling (VAC) analysis as it overcomes some of the limitations of traditional non-invasive methods’ used to calculate the ratio of arterial to ventricular end‐systolic elastance (Ea/Ees) [43]. The VAC impersonates an essential task in cardiac and aortic mechanics [43, 44]. The VAC evaluation has an independent diagnostic and prognostic value and can be used to refine risk stratification and monitor therapeutic interventions [43]. Cardiac arrest via myocardial hypoxia induces myocardial edema and therefore increased myocardial stiffness, thus changing Ea/Ees ratio and VAC, respectively. Therefore, the non-invasive GWI and VAC assessment might be a valuable tool in the acute phase to guide the pharmacological management of these patients.

### Limitations

The small sample size of the study limits the significance of our analysis as it was not adequately powered for multiple comparisons. However, from an ethical perspective, the downsizing of experimental groups is part of the 3R strategy. The TEE measurements in swine are challenging due to specific anatomic features. Special attention was paid to avoid foreshortening of the left ventricle in echocardiography, and ante- and retroflexion of the tip was used to correctly identify the apex of the heart. Despite all the precautions, foreshortened ventricle views might have underestimated the left ventricular volumes including the stroke volume, RV D_3_ and RV areas. 3D-TEE is believed to overcome these limitations and would have led to a more precise calculation of the derived parameters. The image quality of two-chamber-views can be limited due to swine anatomy (the bronchus partially conceals the heart from the esophagus). There are no studies comparing RV PS-loops with invasive PV-loops, to the best of our knowledge, due to lack of specific software for RV myocardial work assessment. We used the commercially available LV software provided by GE for RV myocardial work measurements. Therefore, the results must be interpreted with caution. The peripheral arterial pressure was used for PS-loop evaluation, which might be lower than the central arterial due to a lack of pressure augmentation.

## Conclusions

During resuscitation from cardiac arrest, iCPR provides sufficient unloading and preservation of end-organ perfusion by maintaining cardiac output and improving myocardial work recovery. The addition of inhaled nitric oxide enables improved preservation of RV function as determined by better recovery of RV global work indices. Myocardial work estimation by echocardiographic analysis can help understand and guide both left and right ventricular unloading to minimize myocardial stress and oxygen demand, while maintaining sufficient end-organ perfusion.

## Data Availability

The datasets used or analyzed during the current study are available from the corresponding author on reasonable request.

## Abbreviations

CA: Cardiac arrest
CO_PWD_: Cardiac output as measured by pulsed-wave Doppler
CO_TD_: Cardiac output measured with thermodilution method using Swan-Ganz catheter
DBP: Diastolic blood pressure
DPAP: Diastolic pulmonary artery pressure
GCW: Global constructed myocardial work
GLS: Global longitudinal strain
GWE: Global myocardial work efficiency
GWI: Global myocardial work index;
GWW: Global wasted work
HR: Heart rate
HR_*pp-VF*_ min^.^^−^^1^: Peak to peak inspiration frequency as the heart rate during cardiac arrest
iCPR: Percutaneous mechanical cardiopulmonary resuscitation
iNO: Inhaled nitric oxide
LAX: Apical long axes view
LV: Left ventricle
LVEDV: Left ventricular end-diastolic volume
LVEF: Left ventricular ejection fraction
LVEDP: Left ventricular diastolic pressure
LVOT: Left ventricular outflow tracts
PSL: Pressure strain loop
PV-loop: Pressure–volume loop
PWD: Pulsed-wave Doppler
ROSC: Return of spontaneous circulation
RV: Right ventricle
RVEDD: Right ventricular end-diastolic diameter
RVEDP: Right ventricular diastolic pressure
RVD1: Right ventricle basal diameter
RVD2: Mid right ventricle diameter
RV3: Longitudinal right ventricle diameter
RV-FAC: Right ventricular fractional area change
RVOT: Right ventricular outflow tracts
SBP: Systolic blood pressure
SPAP: Systolic pulmonary artery pressure
STE: 2-D speckle-tracking echocardiography
TASV: Tricuspid annular systolic velocity
TDI: Tissue Doppler imaging
TEE: Transesophageal echocardiography
VAC: Ventricular-arterial coupling
VTI: Velocity time integrals
